# Monocyte-lymphocyte ratio as a predictor of 3-month mortality in elderly heart failure patients: a retrospective Chinese cohort study

**DOI:** 10.3389/fcvm.2025.1665183

**Published:** 2025-09-11

**Authors:** Xiao-Shan Guo, Chong-Xu Wang, Hong-Ju Jiang, Jing Zhu, Jian Wang

**Affiliations:** ^1^ Department of Critical Care Medicine, The Second Affiliated Hospital of Shandong University of Traditional Chinese Medicine, Jinan, China; ^2^ Department of Cardiovascular Medicine, The Second Affiliated Hospital of Shandong University of Traditional Chinese Medicine, Jinan, China

**Keywords:** monocyte-lymphocyte ratio, prognosis, heart failure, China, elderly patients

## Abstract

**Background:**

Heart failure (HF) represents the terminal phase of cardiovascular disease and is the primary cause of mortality in elderly patients diagnosed with HF. Precise early prediction of HF onset and progression is crucial for enhancing survival rates in patients. Central to HF's pathophysiology is inflammation, with the monocyte-lymphocyte ratio (MLR) emerging as a potential novel inflammatory marker. The relationship between MLR and HF in the elderly is not well-defined. Therefore, this study, utilizing the 2016–2019 Sichuan Zigong Heart Failure Database, aimed to explore the correlation between MLR levels and 3-month mortality in elderly HF patients within the Chinese population.

**Methods:**

A retrospective cohort study was conducted using the 2016–2019 Heart Failure Database from Zigong City, Sichuan Province, China. HF was identified based on the diagnostic criteria of the European Society of Cardiology. The MLR was calculated as monocyte count divided by lymphocyte count. Both lymphocyte and monocyte counts were sourced directly from laboratory datasets. Cox regression analysis was performed to assess the relationship between MLR and 3-month mortality, with stratified evaluations conducted based on age, gender, and comorbidity index.

**Results:**

Of the 1,448 elderly HF patients assessed, multivariate regression analyses revealed that the high-level MLR group had a heightened occurrence of 3-month mortalities, presenting a hazard ratio (HR) and a 95% CI of 3.31 (1.42–7.7). In the subgroup analyses, the effect sizes of MLR remained consistent across all subgroups (all *P-*values > 0.05).

**Conclusion:**

MLR is significantly associated with 3-month mortality rates in elderly HF patients. Early MLR evaluations might offer a pathway to augment the life quality and survival outcomes of these patients.

## Background

Heart failure (HF), the terminal phase of cardiovascular disease, is a prevalent mortality cause among the elderly (1). Globally, it impacts nearly 40 million individuals. Annually, heart failure is the cause of death for 10% to 30% of affected patients (2). Factors such as population growth and aging have led to a consistent increase in heart failure patients (3). Early identification of mortality risk in patients with heart failure can substantially enhance their life quality and survival chances.

Several factors have been identified that correlate with mortality rates in patients with HF. For instance, Grodin et al., analyzing data from TOPCAT, deduced that diminished serum chloride levels independently elevate cardiovascular and all-cause mortality risk in HF patients with preserved ejection fraction (HFpEF) (4). However, this study only included HF patients with standard ejection fraction values, implying that the findings can't be generalized to all HF patients with low chloride levels. Beltowski et al. investigated the relationship between microalbuminuria and mortality in acute HF patients. They classified 426 HF patients who presented at the emergency department into three categories based on their left ventricular ejection fraction: the HF patients with preserved ejection fraction (HFpEF) group, HF with mildly reduced ejection fraction (HFmEF) group, and the HF with reduced ejection fraction (HFrEF) group. They found that AURC only prognosticated outcomes for patients in the HFmEF and HFrEF groups (5). Nonetheless, this study's limited sample size and the focus solely on acute heart failure patients restrict its broader clinical applicability. Therefore, identifying a more stable and readily available index to gauge mortality risk is imperative for optimizing elderly HF patient care.

Inflammation plays a pivotal role in the pathogenesis of HF and is a central process in its pathophysiology. Previous studies show that inflammatory markers, such as C-reactive protein (CRP), tumor necrosis factor (TNFα), and interleukin (IL-6), contribute to cardiovascular system adaptability during the early phases of HF (6, 7). However, these markers exacerbate HF's advanced stages by promoting myocardial fibrosis. These findings underline the detrimental effects of elevated pro-inflammatory cytokines on outcomes and cardiac remodeling in HF patients. Therefore,we hypothesized that MLR holds significant implications for the development and progression of HF, particularly in elderly patients. Yet, the relationship between MLR and HF in the elderly remains to be elucidated. Therefore, this study investigated the clinical relevance and prognostic value of MLR in elderly patients with HF.

## Methods

### Study population

This study sourced data from a single-center database available on PhysioNet, which collated details on 2008 adult HF patients. These patients were admitted to the Fourth People's Hospital of Zigong City, Sichuan Province, China, between December 2016 and June 2019. The primary aim was to delineate the characteristics of the Chinese HF population. The dataset originates from the first laboratory test results upon patient admission, including demographic data, baseline clinical characteristics, comorbidities, laboratory findings, prescribed medications, and outcome data. Adhering to the STROBE (Strengthening the Reporting of Observational Studies in Epidemiology) guidelines, this investigation aimed to discern if a heightened MLR level corresponded to an escalated mortality risk. The Ethics Committee of the Fourth People's Hospital of Zigong City approved this study (approval number: 2020-010). Given the retrospective design of this study, the necessity for informed consent was exempted. All research processes rigorously complied with the Declaration of Helsinki. HF diagnosis adhered to the criteria of the European Society of Cardiology.

### Study variables

The exposure variable in this study was the MLR, with the primary outcome being the risk of mortality within three months (all-cause mortality). Supplementary Data mined from the database encompassed variables like age, gender, Charlson Comorbidity Index (CCI) score, New York Heart Association (NYHA) cardiac classification, systolic blood pressure, body mass index, history of previous myocardial infarction, history of congestive heart failure, diabetes mellitus, chronic kidney disease, brain natriuretic peptide levels, creatinine, urea, hemoglobin levels, hematocrit, and levels of calcium, potassium, sodium, chloride, and pH.

### Statistical analysis

Categorical variables were presented as percentages, while continuous variables were denoted as mean ± standard deviation. Patients were bifurcated into two groups according to their MLR values. Initially, linear regression models coupled with chi-square tests were employed to compare the baseline characteristics between groups. Subsequently, univariate and multivariate Cox proportional-hazards regression analyses were conducted to ascertain the association between MLR and the 3-month mortality risk. The models were formulated as follows: The first, a minimally-adjusted model, accounted for age and sex. The second model, the minimum-corrected model, is factored in confounders such as the Charlson Comorbidity Index (CCI) score and the New York Heart Association (NYHA) cardiac function class. This model was refined by incorporating the brain natriuretic peptide as an additional parameter. Covariates for adjustment were judiciously chosen, predicated on their potential to modify the regression coefficients by a minimum of 10%. Both 95% confidence intervals (CI) and hazard ratios (HR) were computed for all models. Furthermore, a sensitivity analysis was instituted to validate the robustness of the results. Stratified analyses and interactions were conducted based on age, gender, and CCI score. The dose-response relationship between MLR and 3-month mortality risk was evaluated using a smoothed curve fitting via the penalized spline method. Cumulative 3-month mortality risk across the groups was analyzed utilizing Kaplan–Meier (KM) curves. All tests were two-sided, and a *P*-value < 0.05 was deemed statistically significant. Data analyses were performed using the R statistical package (version 3.6.3, R Foundation for Statistical Computing, Vienna, Austria) and the free statistical software version 1.8.

## Results

### Patient selection

**Figure 1:**
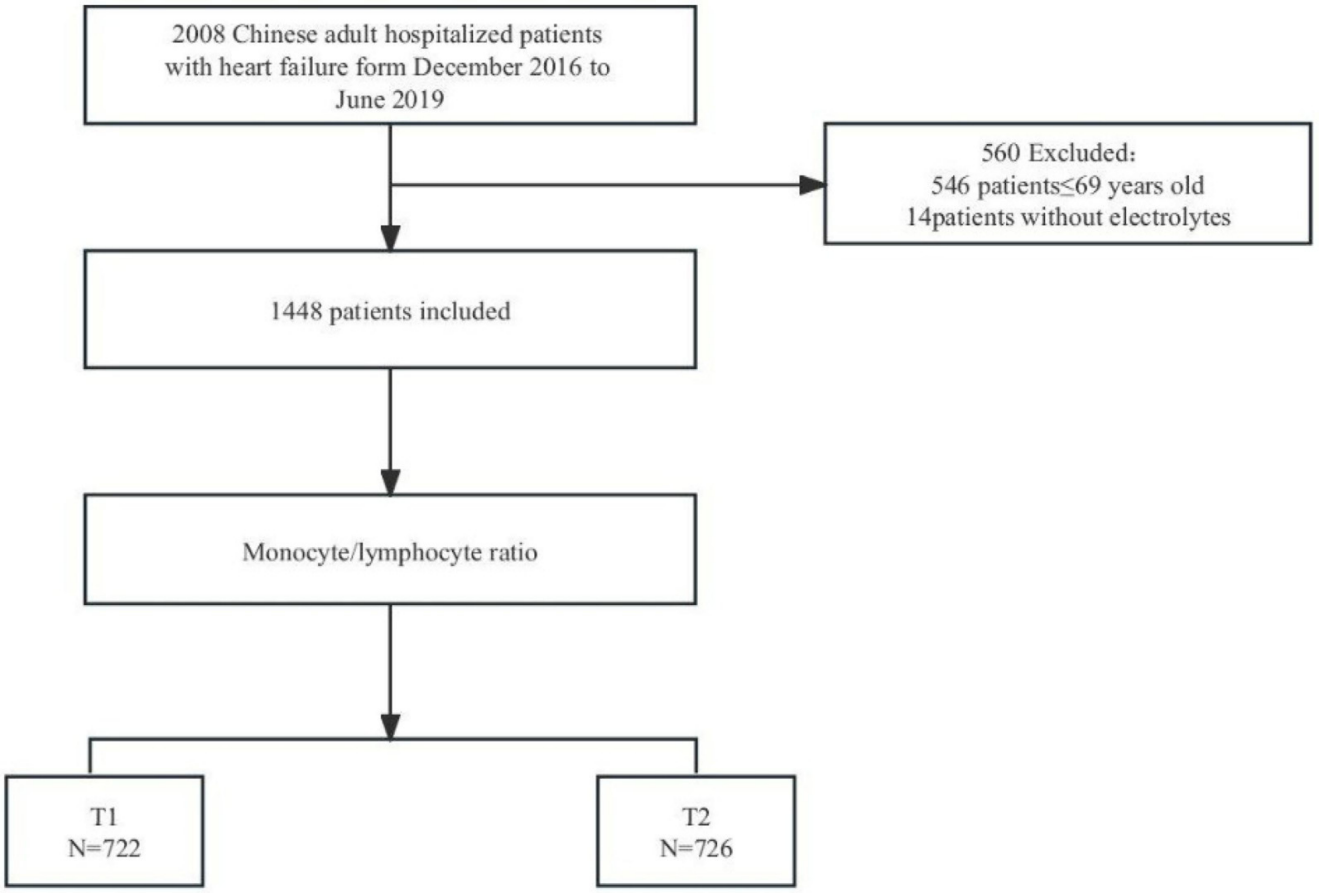
Flowchart of study cohort.T1: Low level of monocyte-lymphocyte ratio: 0.026-0.475; T2:high level of monocyte-lymphocyte ratio: 0.476-6.65.

From a database of 2,008 patients with HF, 546 were excluded due to being aged ≤ 69. An additional 14 were removed due to missing monocyte and lymphocyte data, leaving 1,448 patients for analysis (Figure 1).

### Baseline characteristics

Table 1 outlines the attributes of the analyzed patients. Of the 1,448 patients (all aged 70 or above), 51.1% were over 80, and 38.7% were males. Compared to the low MLR group, individuals in the high MLR group had a higher propensity to be male and exhibited increased CCI scores, NYHA classifications, creatinine, urea, and brain natriuretic peptide levels. Conversely, they displayed reduced body mass index, systolic blood pressure, hemoglobin concentration, chloride, and sodium values.

**Table 1:** Baseline characteristics according to categories of MLR ratio.

Variables	Total (*n* = 1448)	T1 (*n* = 722)	T2 (*n* = 726)	*p*
Male	560 (38.7)	232 (32.1)	328 (45.2)	<0.001
Age, year				<0.001
<80	708 (48.9)	429 (56.1)	279 (40.8)	
≥80	740 (51.1)	336 (43.9)	404 (59.2)	
SBP, mmHg	133.2 ± 24.3	134.3 ± 24.0	132.1 ± 24.5	0.081
NYHA cardiac function classification				0.011
Class II	247 (17.1)	133 (18.4)	114 (15.7)	
Class III	762 (52.6)	396 (54.8)	366 (50.4)	
Class IV	439 (30.3)	193 (26.7)	246 (33.9)	
Previous MI	126 (8.7)	57 (7.9)	69 (9.5)	0.277
Previous CHF	1,341 (92.6)	658 (91.1)	683 (94.1)	0.032
Diabetes	337 (23.3)	171 (23.7)	166 (22.9)	0.712
Chronic kidney disease	375 (25.9)	160 (22.2)	215 (29.6)	0.001
CCI score ≥ 3	382（26.5）	157（21.8）	225（31）	< 0.001
Hemoglobin (g/L)	111.2 ± 23.5	113.5 ± 22.4	108.9 ± 24.4	<0.001
Hematocrit (%)	0.3 ± 0.1	0.3 ± 0.1	0.3 ± 0.1	<0.001
Calcium (mmol/L)	2.3 ± 0.2	2.3 ± 0.2	2.3 ± 0.2	0.104
Potassium (mmol/L)	4.0 ± 0.7	4.0 ± 0.6	4.1 ± 0.8	0.013
Chloride (mmol/L)	102.0 ± 6.1	103.3 ± 5.1	100.7 ± 6.6	<0.001
Sodium (mmol/L)	138.3 ± 5.0	139.4 ± 4.2	137.2 ± 5.5	<0.001
BMI (Kg/m^2^)	20.7 (18.4, 23.4)	20.8 (18.5, 23.8)	20.3 (18.3, 22.7)	0.006
Creatinine (mmol/L)	92.0 (67.2, 129.1)	86.1 (63.7, 117.4)	100.8 (70.2, 140.5)	<0.001
Urea (mmol/L)	8.4 (6.0, 12.1)	7.5 (5.7, 10.9)	9.4 (6.5, 13.5)	<0.001
BNP (pg/ml)	772.4 (330.4, 1,723.1)	711.2 (292.2, 1,595.6)	888.6 (345.8, 1,938.3)	0.003

SBP, systolic blood pressure; MI, myocardial infarction; CHF, congestive heart failure; BMI, body mass index; BNP, brain natriuretic peptide; CCI score, Charlson comorbidity index score. CCI is a widely used tool for assessing the burden of comorbid diseases and their impact on a patient's prognosis, particularly in relation to mortality risk. The CCI assigns weighted scores to various comorbidities, with each score reflecting the severity of the disease and its potential effect on a patient's life expectancy. The total score helps predict the patient's risk of death, which is useful in clinical practice and research.

### Relationship between MLR and 3-month mortality risk

As presented in Table 2, 30 patients (4.3%) succumbed within three months. Analyzing the MLR dichotomous subgroups, 7 (1%) in the lower and 23 (3.2%) in the higher MLR subgroup experienced mortality within the same timeframe. Multivariate Cox proportional-hazard regression analyses substantiated that elevated MLR significantly heightened the 3-month mortality risk. Specifically, the multivariable-adjusted model exhibited an HR of 2.71 (95% CI, 1.13–6.47) for patients in the T2 group relative to those in the T1 group during model 3. All *P*-values for trend tests were below 0.05, endorsing the robustness of our findings.

**Table 2:** MLR and mortality risk at 3-month after discharge in HF.

MLR	Total(n)	Event(%)	Crude HR(95%CI)	Adjusted HR(95%CI)		
				Model 1	Model 2	Model 3
T1	722	7 (1)	1(Ref)	1(Ref)	1(Ref)	1(Ref)
T2	726	23 (3.2)	3.31 (1.42∼7.7)	3.04 (1.29∼7.18)	2.77 (1.16∼6.61)	2.71 (1.13∼6.47)

Model 1 was adjusted for age and sex. Model 2 was adjusted for age, sex, NYHA class and CCI. Model 3 was adjusted for all covariates in model 2 and additionally adjusted for BNP.

### Subgroup analysis

**Figure 2:**
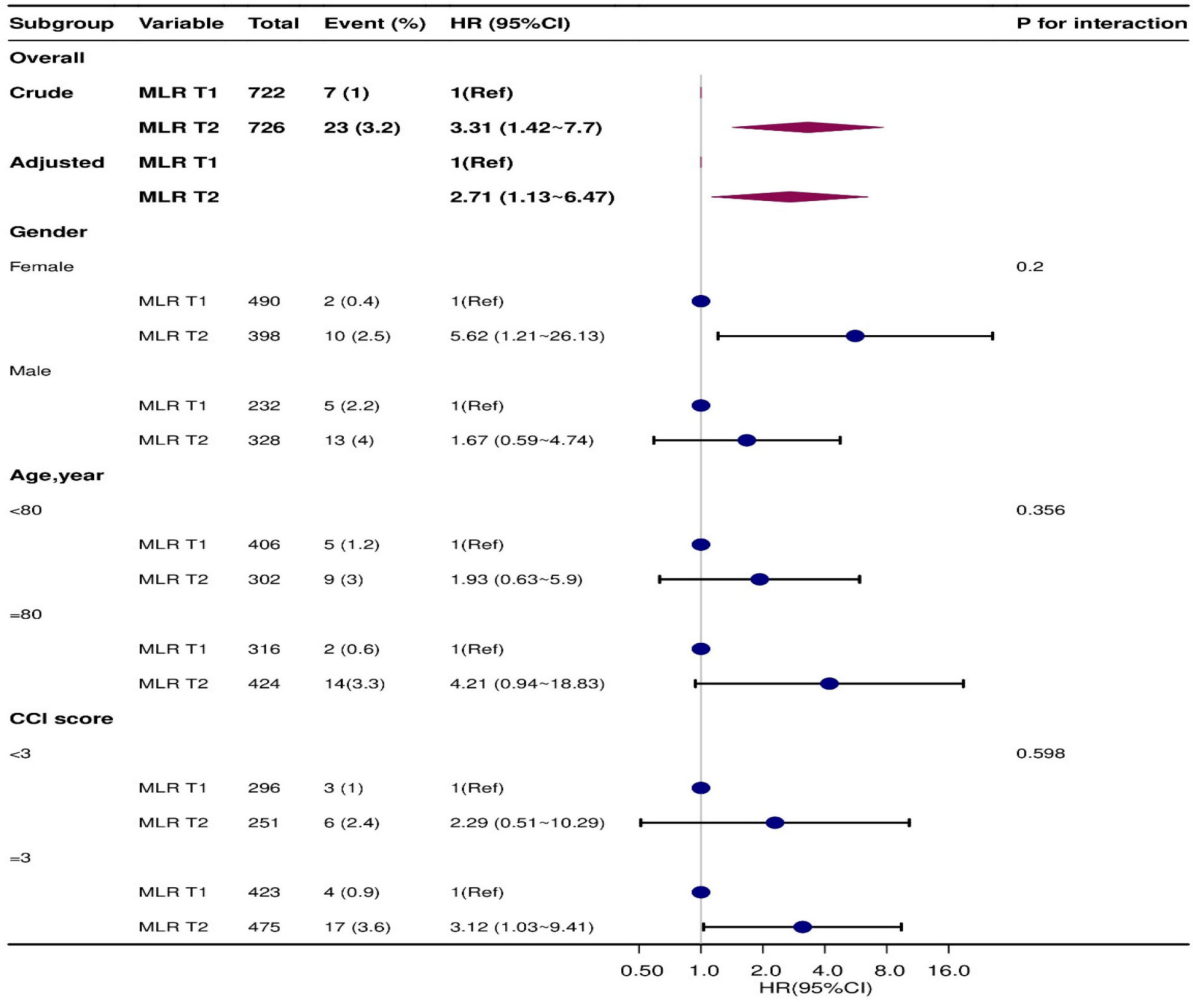
Subgroup analyses for association of MLR categories and 3-month mortality risk (model 3), P for interaction all >0.05.MLR: monocyte-lymphocyte ratio.T1: Low level of monocyte-lymphocyte ratio: 0.026-0.475; T2:high level of monocyte-lymphocyte ratio: 0.476-6.65.

Figure 2 presents the stratification and interaction analysis concerning the relationship between MLR and the 3-month mortality risk in patients with HF. The results of the subgroup analyses were congruent with those obtained from the multivariate Cox regression analyses. Moreover, the interaction analysis revealed no evident interactions within the subgroups.

### Kaplan–Meier survival curve

**Figure 3:**
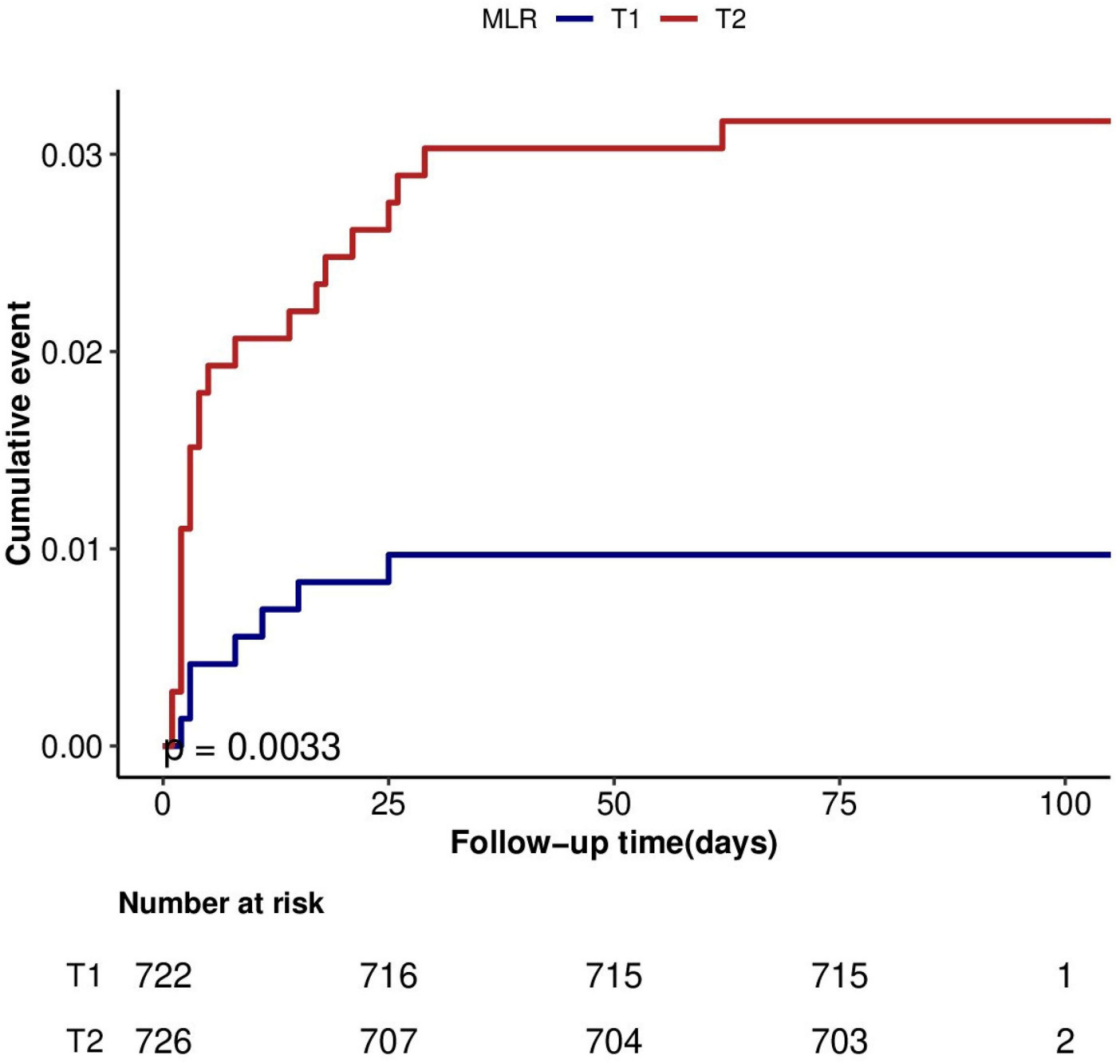
Cumulative hazard of mortality according to categories of MLR at 3-month after discharge in HF.MLR: monocyte-lymphocyte ratio.

As depicted in Figure 3, patients in the T2 group exhibited a notably elevated 3-month mortality risk compared to their counterparts in the T1 group (*P* < 0.01).

## Discussion

Our analysis drew upon data from the Sichuan Zigong Heart Failure Database (8). From our assessment, this study presents a novel observation: a concurrent rise in MLR as HF mortality escalates. We discerned a significant correlation between MLR and 3-month mortality among patients with HF. Once potential confounders were accounted for, MLR emerged as a salient predictor of 3-month mortality within this cohort. These findings underscore the potential utility of MLR as a prognostic tool for both HF progression and 3-month mortality.

The results of this study suggest a positive association between MLR and 3-month mortality in elderly HF patients. To mitigate the effects of potential confounders, we employed three distinct Cox regression models to elucidate the relationship between MLR and 3-month mortality in this demographic. Upon complete adjustments in the model 3, the effect value was established at 3.16 (1.33–7.53). This translates to a 216% elevation in the 3-month mortality risk among elderly HF patients for each unit increment in MLR. Based on prevailing clinical consensus, once adjustments for potential confounders have been made, any effect value alteration of MLR by 10% or greater firmly establishes a potent positive correlation between MLR and 3-month mortality in elderly patients with HF. For a more nuanced sensitivity analysis, we bifurcated MLR into two categories, and the resultant data exhibited both stability and reliability. Furthermore, our subgroup analysis, which factored in variables such as age, gender, and co-morbidity index, displayed consistent results across all subgroups, devoid of any interactions. The K-M survival curves, adjusted per model 3, showcased the differential in the 3-month mortality rate between varying MLR levels among elderly HF patients. The results were consistent with trend validation.

Previous studies have highlighted the mononuclear lymphocyte ratio (MLR) as a pivotal risk determinant for conditions such as pneumonia, cancer, depression, and cardiovascular ailments, especially in its role in the progression and outcome prediction of cardiovascular diseases (9–13). Mirna et al. conducted a retrospective cohort study involving 202 myocarditis patients and revealed MLR to be a more salient factor concerning hospitalization duration than conventional inflammatory markers like CRP, white blood cell count, IL-6, or calcitonin gene (14). Gijsberts et al. identified a correlation between elevated MLR and increased levels of NT-proBNP in patients undergoing coronary arteriography, citing it as an independent predictor for heart failure patients’ readmission after an average follow-up of 1.3 years (15). Nevertheless, the prognostic implications of MLR for elderly Chinese HF patients remain underexplored. Hence, our analysis aimed to bridge this knowledge gap. Aligning with our conclusions, prior studies have ascertained that an admission-time MLR is linked with a multivariate-adjusted risk of readmission or mortality. A retrospective cohort study involving 678 patients diagnosed with non-ST-segment elevation myocardial infarction (NSTEMI) who underwent percutaneous coronary intervention (PCI) in Beijing, China, established that heightened MLR levels independently forecasted long-term major adverse cardiac events (MACE) for patients with NSTEMI (16). Zhai et al. observed that, among patients in a cardiac intensive care unit, those in the highest MLR quartile group exhibited the steepest mortality rates (7.8% in the lowest group vs. 16.3% in the highest group, *p* < 0.001) (6). Oh et al. highlighted that in patients with chronic kidney disease (CKD), the high MLR cohort faced an escalated risk for composite cardiovascular disease (CVD) events, inclusive of cardiovascular disease onset, heart failure, CVD mortality, and all-cause mortality, with an HR of 1.27 (95% CI, 1.1–1.48) (7). Delcea et al. juxtaposed the neutrophil-lymphocyte ratio (NLR), monocyte-lymphocyte ratio (MLR), and platelet-lymphocyte ratio (PLR) to discern the most clinically pertinent metrics for forecasting hospitalization outcomes in heart failure patients (17). Their results pinpointed all three metrics as independent mortality predictors in such patients but uniquely distinguished MLR as an independent prognosticator for in-hospital mortality (HR 1.68, 95% CI 1.22–2.32, *p* = 0.002). Another study investigated 171 heart failure (HF) patients, primarily examining the prognostic value of NLR,MLR,and LMR in predicting major adverse cardiovascular events (MACE) and mortality across different LVEF categories.Their findings demonstrated that patients with HFrEF and HFmrEF exhibited significantly higher NLR and MLR levels but lower LMR levels. While MACE incidence was similar across groups, the HFrEF group had a significantly higher mortality rate (18). The evidence suggests a potential correlation between MLR and heart failure. Consequently, we hypothesized that a reduction in MLR levels might lead to a decline in 3-month mortality among heart failure patients, presenting a pioneering avenue to enhance their health outcomes potentially. However, it's imperative that additional prospective studies be conducted to verify if these findings hold true across a more extensive population.

There are some aspects worth mentioning in the present study compared with previous studies. Since the Sichuan Zigong Heart Failure Database only includes the Chinese population, previous studies have remained uncharted regarding the relationship between MLR and 3-month mortality in Chinese patients with heart failure. For a more graphic depiction of this relationship amongst elderly heart failure patients, we crafted a K-M survival curve.

Also, this study has some limitations. Even as our results signify a positive correlation of MLR with 3-month mortality in elderly patients with heart failure, the retrospective nature of our cohort study inherently introduces biases. Hence, imminent prospective evaluations are requisite to clarify this relationship. Additionally, as the Sichuan Zigong Heart Failure Database is localized to the Chinese population, generalizing our findings about MLR and 3-month mortality in older heart failure patients to other populations remains tentative.First, healthcare-access patterns in China may differ from European cohorts universal-coverage systems. Second, ethnicity-specific factors like dietary sodium intake or ACEI prescription rates could influence leukocyte indices' prognostic utility.While the inflammatory mechanism linking MLR to mortality may be universal, the optimal prognostic thresholds require age- and ethnicity-specific calibration (19) Furthermore,the lack of data on medication dosages and the important variable of left ventricular ejection fraction (LVEF) in this database may impact the study results, despite our comprehensive assessment of the clinical condition of heart failure patients using other key indicators.The prognostic value of MLR may vary by heart failure subtypes: inflammatory markers such as MLR may play a dominant role in HFrEF (LVEF ≤ 40%), whereas diastolic dysfunction might be more critical in HFpEF. Failure to stratify by LVEF could obscure these subtype-specific associations (18). The lack of LVEF data may introduce confounding, as patients with reduced vs. preserved LVEF have distinct risk profiles. However, the inclusion of BNP in our multivariate model may partially mitigate this, as it correlates with EF and overall HF severity (20). The lack of medication data (beta-blockers and RAAS inhibitors) represents a key limitation, as these therapies are known to modify mortality risk in heart failure. Their potential confounding effects cannot be excluded.Future prospective studies should systematically capture medication data and ejection fraction (EF) values to better control for this confounder.

## Conclusion

The findings from this retrospective cohort study indicate that the MLR at admission was directly correlated with the 3-month mortality risk in elderly patients with HF. MLR serves as a straightforward yet productive inflammatory marker that can be routinely detected in clinical settings, warranting the scrutiny of healthcare practitioners. However, the precise underlying mechanism and its applicability to diverse populations remain to be elucidated. It is imperative to undertake randomized controlled trials with larger sample sizes to furnish robust evidence for clinical practice guidelines.

## Data Availability

The datasets presented in this study can be found in online repositories. The names of the repository/repositories and accession number(s) can be found in the article/Supplementary Material.

## References

[1] Adamo L Rocha-Resende C Prabhu SD Mann DL. Reappraising the role of inflammation in heart failure [published correction appears in nat rev cardiol. 2021;18(10):735]. Nat Rev Cardiol. (2020) 17(5):269–85. 10.1038/s41569-019-0315-x 31969688

[2] Sabbatini AR Kararigas G. Menopause-related estrogen decrease and the pathogenesis of HFpEF: JACC review topic of the week. J Am Coll Cardiol. (2020) 75:1074–82. 10.1016/j.jacc.2019.12.049 32138968

[3] Groenewegen A Rutten FH Mosterd A Hoes AW. Epidemiology of heart failure. Eur J Heart Fail. (2020) 22(8):1342–56. 10.1002/ejhf.1858 32483830 PMC7540043

[4] Grodin JL Testani JM Pandey A Sambandam K Drazner MH Fang JC Perturbations in serum chloride homeostasis in heart failure with preserved ejection fraction: insights from TOPCAT. Eur J Heart Fail. (2018) 20(10):1436–43. 10.1002/ejhf.1229 29893446

[5] Alatas ÖD Biteker M Demir A Yildirim B Acar E Gökçek K Microalbuminuria and its prognostic significance in patients with acute heart failure with preserved, mid-range, and reduced ejection fraction. And reduced ejection fraction. Microalbuminúria e seu Significado Prognóstico em Pacientes com Insuficiência Cardíaca Aguda com Fração de Ejeção Preservada, Intermediária e Reduzida. Arq Bras Cardiol. (2022) 118(4):703–9. 10.36660/abc.20201144 35137781 PMC9007018

[6] Zhai G Liu Y Wang J Zhou Y. Association of monocyte-lymphocyte ratio with in-hospital mortality in cardiac intensive care unit patients. Int Immunopharmacol. (2021) 96:107736. 10.1016/j.intimp.2021.107736 34162134

[7] Oh ES You Z Nowak KL Jovanovich AJ. Association of monocyte count and monocyte/lymphocyte ratio with the risk of cardiovascular outcomes in patients with CKD. Kidney360. (2022) 3(4):657–65. 10.34067/KID.0007922021 35721602 PMC9136887

[8] Zhang Z Cao L Chen R Zhao Y Lv L Xu Z Electronic healthcare records and external outcome data for hospitalized patients with heart failure. Sci Data. (2021) 8(1):46. 10.1038/s41597-021-00835-9 33547290 PMC7865067

[9] Damar Çakırca T Torun A Çakırca G Portakal RD. Role of NLR, PLR, ELR, and CLR in differentiating COVID-19 patients with and without pneumonia. Int J Clin Pract. (2021) 75(11):e14781. 10.1111/ijcp.14781 34482573 PMC8646493

[10] Kang Y Zhu X Lin Z Zeng M Shi P Cao Y Compare the diagnostic and prognostic value of MLR, NLR, and PLR in CRC patients. Clin Lab. (2021) 67(9). 10.7754/Clin.Lab.2021.201130 34542964

[11] Meng F Yan X Qi J He F. Association of neutrophil to lymphocyte ratio, platelet to lymphocyte ratio, and monocyte to lymphocyte ratio with depression: a cross-sectional analysis of the NHANES data. J Affect Disord. (2022) 315:168–73. 10.1016/j.jad.2022.08.004 35932936

[12] Wang Z Qin Y Chai X Lu L Xue P Lu R Systemic inflammatory biomarkers predict survival of patients treated with tyrosine kinase inhibitors for metastatic renal cell carcinoma. Cancer Control. (2023) 30:10732748231197511. 10.1177/10732748231197511 37673428 PMC10486224

[13] Aydın C Engin M. The value of inflammation indexes in predicting patency of saphenous vein grafts in patients with coronary artery bypass graft surgery. Cureus. (2021) 13(7):e16646. 10.7759/cureus.16646 34462681 PMC8387011

[14] Gijsberts CM Ellenbroek GHJM Ten Berg MJ Huisman A van Solinge WW Lam CS Effect of monocyte-to-lymphocyte ratio on heart failure characteristics and hospitalizations in a coronary angiography cohort. Am J Cardiol. (2017) 120(6):911–6. 10.1016/j.amjcard.2017.06.020 28779870

[15] Mirna M Schmutzler L Topf A Hoppe UC Lichtenauer M. Neutrophil-to-lymphocyte ratio and monocyte-to-lymphocyte ratio predict the length of hospital stay in myocarditis. Sci Rep. (2021) 11(1):18101. 10.1038/s41598-021-97678-6 34518607 PMC8438016

[16] Fan Z Li Y Ji H Jian X. Prognostic utility of the combination of monocyte-to-lymphocyte ratio and neutrophil-to-lymphocyte ratio in patients with NSTEMI after primary percutaneous coronary intervention: a retrospective cohort study. BMJ Open. (2018) 8(10):e023459. 10.1136/bmjopen-2018-023459 30341133 PMC6196857

[17] Delcea C Buzea CA Vijan A Draghici A Stoichitoiu LE Dan GA. Comparative role of hematological indices for the assessment of in-hospital outcome of heart failure patients. Scand Cardiovasc J. (2021) 55(4):227–36. 10.1080/14017431.2021.1900595 33761824

[18] Bećirović E Bećirović M Ljuca K Babić M Bećirović A Ljuca N The inflammatory burden in heart failure: a cohort study on potential biomarkers in heart failure with reduced and mildly reduced ejection fraction. Cureus. (2025 Mar 6) 17(3):e80159. 10.7759/cureus.80159 40190877 PMC11972061

[19] Zhang Y Feng L Zhu Z He Y Li X. Association between blood inflammatory indices and heart failure: a cross-sectional study of NHANES 2009–2018. Acta Cardiol. (2024) 79(4):473–85. 10.1080/00015385.2024.2356325 38771356

[20] Januzzi JL van Kimmenade R Lainchbury J Bayes-Genis A Ordonez-Llanos J Santalo-Bel M NT-proBNP testing for diagnosis and short-term prognosis in acute destabilized heart failure: an international pooled analysis of 1256 patients: the international collaborative of NT-proBNP study. Eur Heart J. (2006) 27(3):330–7. 10.1093/eurheartj/ehi631 16293638

